# Novel Molecular Characterization of Colorectal Primary Tumors Based on miRNAs

**DOI:** 10.3390/cancers11030346

**Published:** 2019-03-11

**Authors:** Elisa Conde, Alejandro Pascual, Daniel Prieto-Cuadra, Val F. Laza, Javier Molina-Cerrillo, Miren Edurne Ramos-Muñoz, Esperanza Macarena Rodríguez-Serrano, José Luis Soto, Alfredo Carrato, María Laura García-Bermejo, Carmen Guillén-Ponce

**Affiliations:** 1 Biomarkers and Therapeutic Targets Group and Core Facility, Ramon y Cajal Research Institute, (IRYCIS), 28034 Madrid, Spain; edurneramosmunoz@gmail.com (M.E.R.-M.); e.makarena@hotmail.es (E.M.R.-S.); 2 Pathology Department, Ramon y Cajal Research Institute, University Hospital, 28034 Madrid, Spain; alejandro.pascual@salud.madrid.org; 3 SynlabPathology, Pathology Department, Virgen de la Victoria, University Hospital, 29010 Málaga, Spain; dannielpriet@hotmail.com; 4 Microbiology Department and Bioinformatics Core Facility, IRYCIS, 28034 Madrid, Spain; valfernandez.vf@gmail.com; 5 Medical Oncology Department, Ramon y Cajal Research Institute, University Hospital, IRYCIS, 28034 Madrid, Spain; javier.molinace@gmail.com (J.M.-C.); carmenguillenponce@gmail.com (C.G.-P.); 6 Hereditary Cancer Program Valencian Region, Molecular Genetics Laboratory, Elche University Hospital, Elche, 03202 Alicante, Spain; soto_jos@gva.es; 7 Medical Oncology Department, Ramon y Cajal Research Institute, University Hospital, IRYCIS, Alcala University, 28034 Ciberonc, Spain; acarrato@telefonica.net

**Keywords:** miRNAs, colorectal cancer, patient stratification, biomarkers

## Abstract

microRNAs (miRNA) expression in colorectal (CR) primary tumours can facilitate a more precise molecular characterization. We identified and validated a miRNA profile associated with clinical and histopathological features that might be useful for patient stratification. In situ hybridization array using paraffin-embedded biopsies of CR primary tumours were used to screen 1436 miRNAs. 17 miRNAs were selected for validation by quantitative reverse transcription polymerase chain reaction (qRT-PCR) (*n* = 192) and were further correlated with clinical and histopathological data. We demonstrated that miRNAs associated to Colorectal Cancer (CRC) diagnosis age (over 50s and 60s) included miR-1-3p, miR-23b-3p, miR-27b-3p, miR-143-3p, miR-145-5p and miR-193b-5p. miR-23b-3p and miR-24-3p discriminated between Lynch Syndrome and sporadic CRC. miR-10a-5p, miR-20a-5p, miR-642b and Let-7a-5p were associated to stroma abundance. miR-642b and Let-7a-5p were associated with to peritumoral inflammation abundance. miR-1-3p, miR-143-3p and miR-145-5p correlated with mucinous component. miR-326 correlated with tumour location (right or left sided). miR-1-3p associated with tumour grade. miR-20a-5p, miR-193b-5p, miR-320a, miR-326 and miR-642b-3p associated to tumour stage and progression. Remarkably, we also demonstrated that miR-1-3p and miR-326 expression significantly associated with patient overall survival (OS). Hierarchical clustering and bioinformatics analysis indicated that selected miRNAs could re-classify the patients and work cooperatively, modulating common target genes involved in colorectal cancer key signalling pathways. In conclusion, molecular characterization of CR primary tumours based on miRNAs could lead to more accurate patient reclassification and may be useful for efficient patient management.

## 1. Introduction

Colorectal cancer (CRC) is the fourth leading cause of cancer death world-wide, with an annual incidence of approximately 1.4 million and causes around 700,000 deaths/year [1]. Even if relevant improvements in patient management has occurred, the mortality of this tumour is still high. Therefore, more accurate tumour characterization and stratification of CRC patients for selection of more appropriate treatments are required.

CRC is a very heterogeneous disease due to clinical history, the pathological features and the molecular mechanisms involved in each case differ. The molecular pathways underlying sporadic CRC include chromosomal instability (CIN) (85% of the cases), microsatellite instability (MSI) and the CpG island methylator phenotype pathway (CIMP). CRC associated with Lynch syndrome is characterized by mutations in the Mismatch repair genes (MMR) (*MSH2*, *MLH1*, *MSH6*, *PMS2*) and *EPCAM* [2,3]. Moreover, different histopathological features like stromal component, histological grade, inflammatory response or mucinous component associated with the tumour also contribute to the heterogeneity of CRC. Indeed, the tumour microenvironment plays a relevant role in the prognosis of the disease [4,5].

CRC has been recently classified, based on molecular and microenvironmental characteristics of the primary tumour, into four molecular subtypes (CMSs): CMS1 (microsatellite instability immune) with hyper- mutations, MSI and strong immune activation; CMS2 (canonical) including an epithelial phenotype with WNT and MYC signalling activation; CMS3 (metabolic) exhibiting epithelial phenotype and metabolic dysregulation; and CMS4 (mesenchymal) characterized by TGFB signalling activation, stromal invasion and angiogenesis [6].

CRC diagnosed patients less than 50 years of age exhibit different histopathological characteristics when compared to older patients [7,8,9]. To date, there are no described molecular bases responsible for these differences and, currently, patient management and treatments are non- age-dependent.

Regarding molecular mechanisms underlying cancer, microRNAs have emerged as critical gene expression regulators [10,11]. MicroRNAs (miRNAs) are negative regulators of target genes involved in cell proliferation, apoptosis and cell migration, among others, therefore acting as onco-miRNAs or tumour suppressor miRNAs [12,13,14]. To date, in CRC, numerous miRNAs have been involved in tumour development, following the Vogelstein model [15,16]. Moreover, different miRNA expression profiles have been identified in primary tumours compared to healthy tissue, exhibiting potential diagnostic value [17]. In particular, miR-31 has been associated with tumour stage and miR-21 with tumour metastasis, highlighting miRNAs as potential prognostic tools. Alterations in miRNAs have also been found to be associated with the mutation status of p53 and KRAS [18] and with the recent tumour molecular subtypes (CMSs) classifications.

All these data demonstrate a role for the involvement of miRNAs in CRC development and progression and strongly suggest that miRNAs could be useful tools for clinical patient management. With this aim and in this work, we have identified and validated in primary CRC tumour biopsies a set of miRNAs associated to different features, including: Location, age, histopathological characteristics, microenvironment characteristics, stage and metastasis, with the final goal of putting forth an easily detectable and useful tool for better patient characterization. Moreover, we have also described the functional significance of these miRNAs in the context of CRC via bioinformatics tools, attempting to identify novel mechanisms underlying CRC development and progression.

## 2. Results

### 2.1. Identification of Tissue miRNAs Differentially Expressed in Colorectal Tumours Based on Age and MMR Defect

In order to identify miRNAs associated with sporadic CRC diagnosed at different ages and associated with MMR defect, we performed a screening experiment for 1436 different miRNAs using hybridization arrays. For this initial experiment, the following paraffin-embedded biopsies were included:

(1) One non-tumour colonic mucosae as a control.
(2) Sporadic CRC-diagnosed patients less than 45 years of age and without Lynch syndrome.
(3) CRC patients with Lynch syndrome.
(4) Sporadic CRC-diagnosed patients over 45 years of age and without Lynch syndrome.

Clinical features of these patients are shown in Table 1.

**Figure 1:**
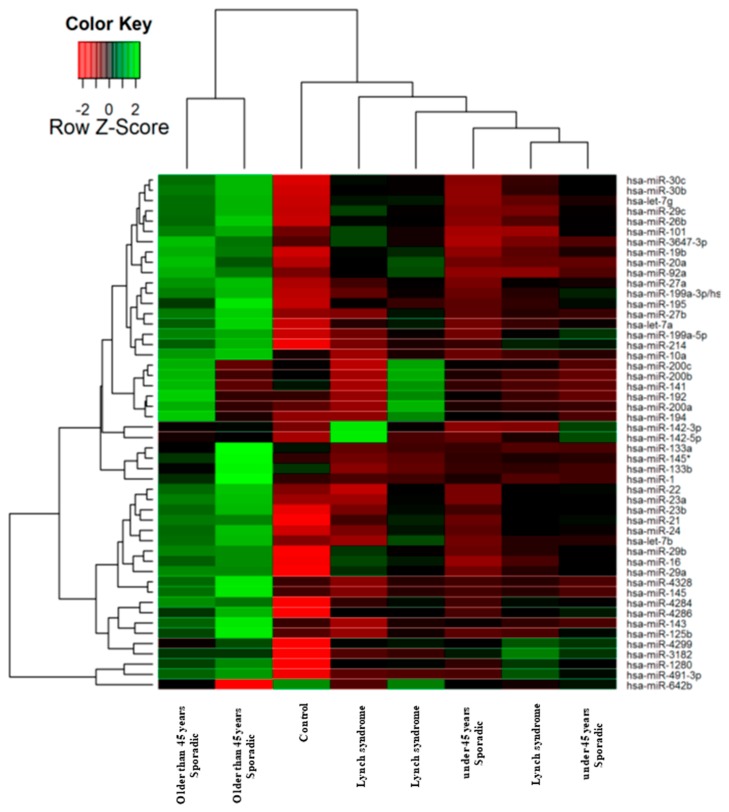
Heat map and hierarchical clustering of microRNAs (miRNAs) in colorectal (CRC) primary tumour biopsies. 1436 miRNAs were analysed in total RNA extracted from paraffin-embedded CRC human biopsies by in situ hybridization. The clustering was performed on all samples, and on the top 50 miRNAs with the highest standard deviation. Normalized log-transformed Hy3 values were used for the analysis.

In Figure 1 we show the heat map diagram of this hybridization array showing the expression of miRNAs organized into a two-way hierarchical clustering, by microRNAs and by samples, presenting the most differentially expressed 50 miRNAs between all samples. This clustering indicates that sporadic CRC-diagnosed patients over 45 years of age exhibited a very different pattern of miRNA expression compared to Lynch and sporadic CRC patients diagnosed before 45 years of age. As expected, miRNA expression in non-tumoral mucosae was different compared to the three patient groups.

### 2.2. Selection of miRNAs for qRT-PCR Validation in a Larger Patient Cohort

In order to provide a reliable miRNA panel potentially useful in clinical practice, we have selected the most promising candidates as follows: (i) Based on technical criteria, miRNAs exhibiting absolute values with a log fold change larger than 1 were initially selected after elimination of unexpressed or inconsistently expressed miRNAs; (ii) based on functional criteria, bioinformatic analysis was performed in order to predict miRNAs with biological significance. Finally, we selected 17 miRNAs (Table 2) for further validation by individual quantitative reverse transcription polymerase chain reaction (qRT-PCR) analysis in 192 new biopsies. Clinical and histopathological information for these patients and the primary tumours used in this validation study are shown in Table 3 and Table 4, respectively.

### 2.3. miRNAs Associated with Sporadic CRC Patient Age at Diagnosis and Discriminate Sporadic CRC vs. Lynch Syndrome Patients

**Figure 2:**
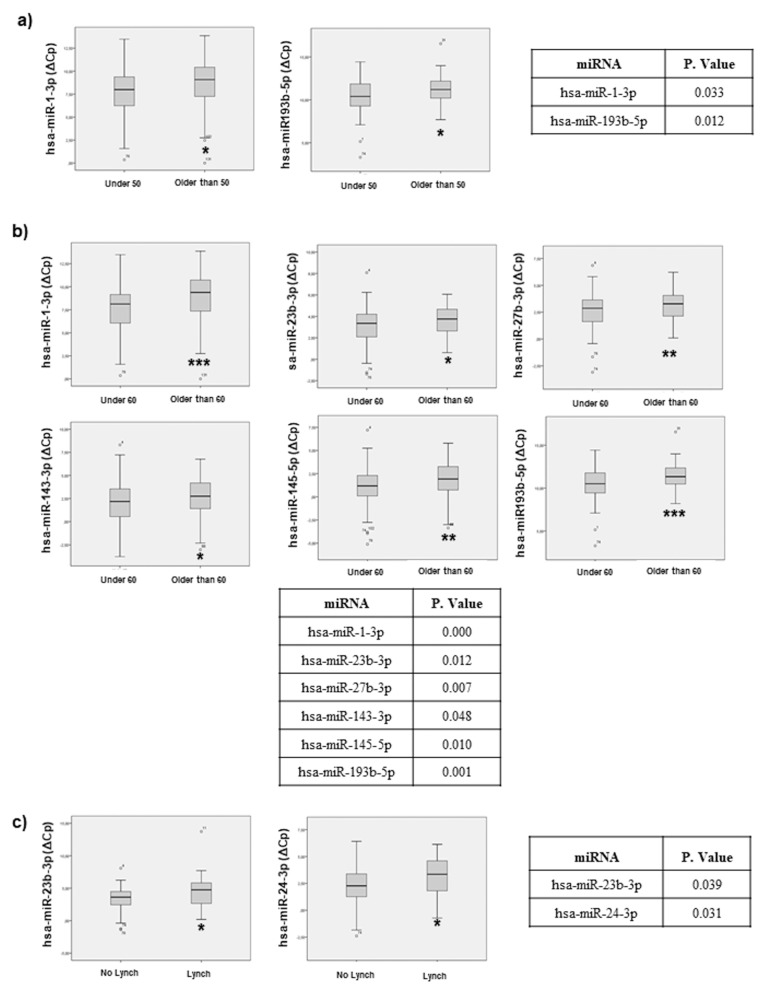
Statistical correlation, of the selected miRNAs, with age and Lynch condition. Statistical analysis using SPSS 19.0 between miRNAs levels expressed as delta crossing threshold (DCT) was performed and data regarding correlation with age (**a**) under 50, (**b**) under 60 and (**c**) Lynch condition are presented. Only miRNAs exhibiting a statistically significant correlation are shown. Data are presented as median and interquartile range with: * *p* < 0.05, ** *p* < 0.01, *** *p* < 0.001.

The 17 selected miRNAs were determined by individual qRT-PCR analysis and expression levels are presented as ΔCq. Statistical analyses demonstrate that miR-1-3p and miR-193b-5p discriminate between diagnosed patients under 50 years of age and diagnosed patients over 50 years of age (Figure 2a). Moreover, a different miRNA expression profile was found between patients diagnosed under 60 years of age and patients diagnosed over 60 years of age. Specifically, miR-1-3p, miR-23b-3p, miR-27b-3p, miR-143-3p, miR-145-5p and miR-193b-5p showed statistical significance across both patient groups (Figure 2b).

These results demonstrate that miRNAs associated with sporadic CRC can be different depending on the age at the time of diagnosis, and these miRNAs might correlate with observed clinical and pathological differences.

Additionally, we have compared ΔCq of the 17 miRNAs between sporadic CRC and Lynch syndrome biopsies. In this comparison, miR-23b-3p and miR-24-3p can discriminate between Lynch Syndrome and sporadic CRC patients (Figure 2c). These results indicate that miRNAs associated with MMR defects can be found and might also contribute to CRC development.

### 2.4. miRNAs Can Be Associated with Histopathological Features in CRC Patients

**Figure 3:**
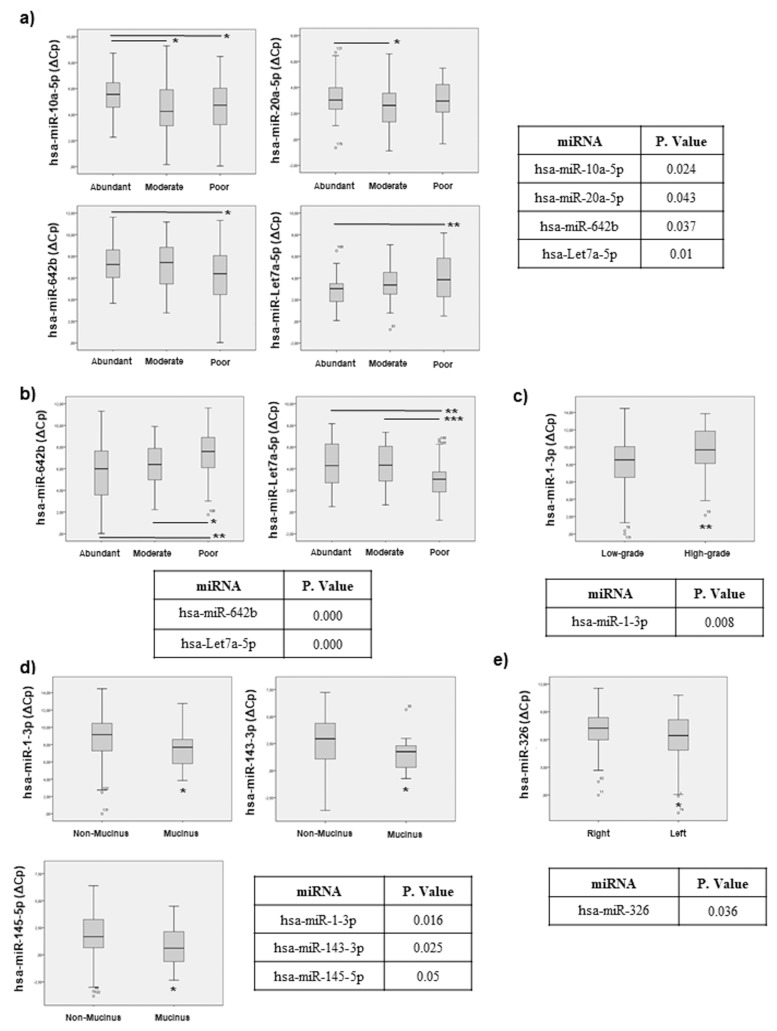
Statistical correlation of the selected miRNAs and biopsy histopathological features. Statistical analysis using SPSS 19.0 between miRNAs levels expressed as DCT was performed and data regarding correlation with: (**a**) stromal component, (**b**) peritumoral inflammatory infiltrate, (**c**) tumour grade, (**d**) mucinous component and (**e**) tumour location, are presented. Only miRNAs exhibiting statistically significant correlations are shown. Data are presented as median and interquartile range with: * *p* < 0.05, ** *p* < 0.01, *** *p* < 0.001.

We also analysed the correlation between our panel of selected miRNAs and the histopathological features available, presented in Table 4 As can be observed in Figure 3a, there was a statistical correlation between some miRNAs and the presence and the abundance of tumour associated stromal component. For example, miR-10a-5p, miR-20a-5p, miR-642b and Let-7a-5p associated to the amount of tumour stroma. Moreover, we have studied the association between these miRNAs and the peritumoral inflammatory infiltrate observed in the primary tumour, demonstrating that miR-642b and Let-7a-5p were differentially expressed depending of the abundance of inflammatory cell infiltrates (Figure 3b).

We have also studied the association between miRNAs and tumour grade, finding that miR-1-3p was differentially expressed between low and high-grade tumours (Figure 3c). Furthermore, we also found that miR-1-3p, miR-143-3p and miR-145-5p showed a statistically significant difference between mucinous and non-mucinous tumours subtypes (Figure 3d).

Finally, our analyses also demonstrated that miR-326 is differentially expressed between left or right tumour localization (Figure 3e).

All these results together validated our selected miRNAs, demonstrating that they associate with different histopathological features of the primary tumour and highlight their potential role as novel molecular mediators in CRC, implying their potential relevance for patient management.

### 2.5. miRNAs Can Indicate CRC Progression: Association to Tumour Staging and Invasion

Tumour staging, including tumour invasiveness, regional lymph node invasion and long-distance metastasis, are fundamental for CRC patient management. Thus, we also studied the relationship between miRNA expression and tumour staging.

**Figure 4:**
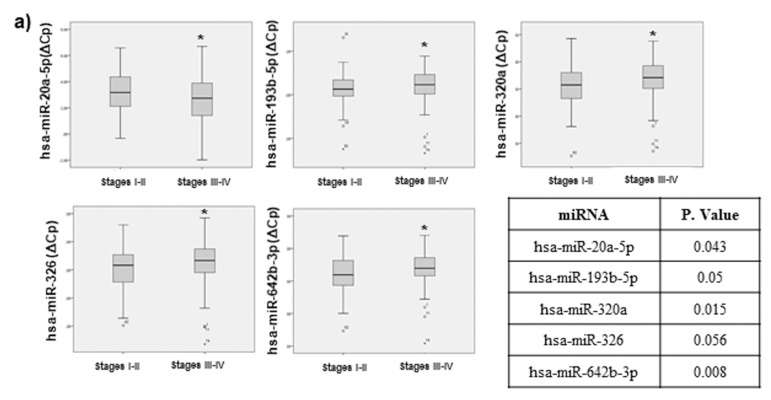

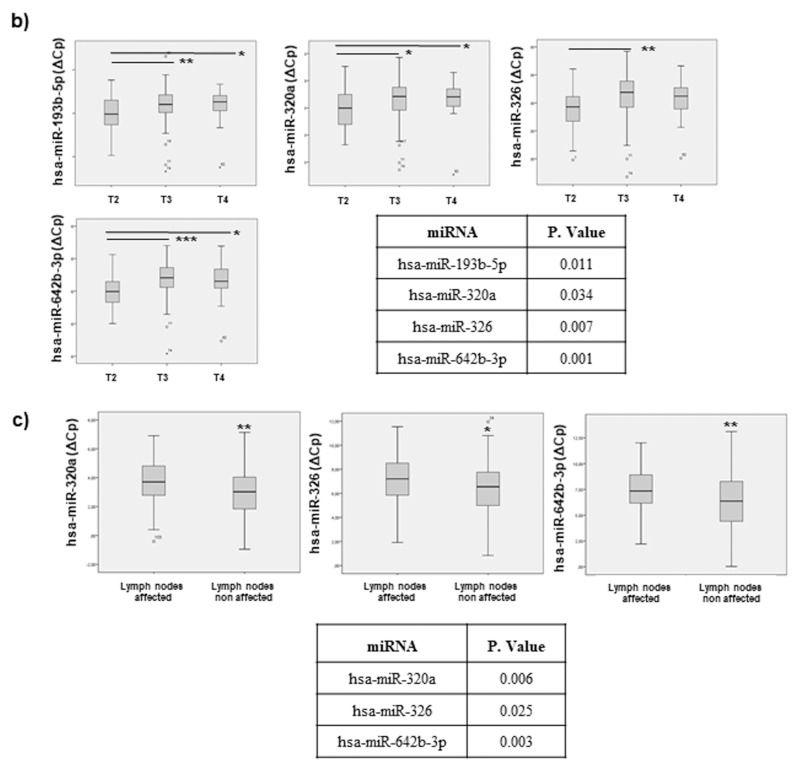
Statistical correlation between selected miRNAs and TNM stage (tumour-node-metastasis TNM staging system), tumour invasion and progression. Statistical analysis using SPSS 19.0 between miRNA levels expressed as DCT was performed. Only miRNAs exhibiting statistically significant correlations are shown. Data are presented as median and interquartile range with *p* values < 0.05. (**a**) data regarding correlation with tumour stage (**b**) tumour invasiveness and (**c**) lymph node affection are presented. * *p* < 0.05, ** *p* < 0.01, *** *p* < 0.001.

Our data demonstrated that 5 miRNAs of our panel exhibited a significant statistical difference among low TNM (tumour-node-metastasis TNM staging system) stages (I–II) and high TNM stages (III–IV): miR-20a-5p, miR-193b-5p, miR-320a, miR-326 and miR-642b-3p (Figure 4a).

When a correlation analysis was performed, including the tumour invasiveness grade (T1–T4), 4 miRNAs exhibited statistical significance: miR-193b-5p, miR-320a, miR-326 and miR-642b-3p (Figure 4b). As can be observed, 4 of the miRNAs that associated with tumour invasiveness were also identified in the tumour stage analysis.

We have also analysed the correlation between our selected miRNAs and data regarding regional lymph nodes affected. Our results demonstrated that three miRNAs: miR-320a, miR-326 and miR-642b-3p were differentially expressed between the presence and absence of lymph nodes affected (Figure 4c). Consistently, these 3 miRNAs also identified in the miRNA profile associated to tumour stage and tumour invasiveness described above.

All these results together not only demonstrate that there is a correlation between our miRNAs and tumour staging and progression, but also highlight some miRNAs that may be responsible for tumour metastasis and could serve as potential useful biomarkers for tumour progression and prognosis.

In summary, 13 out of 17 selected miRNAs were finally validated in the 192 biopsies, exhibiting statistical correlations with patient clinical features.

### 2.6. miRNAs Can Be Useful for Prognosis in CRC: Association with Overall Survival and Progression Free Survival

Patient prognosis is a key clinical parameter for patient management. Therefore, we have study whether miRNAs expression in primary tumour biopsies could also associate with overall survival and progression free survival in our cohort. 

**Figure 5:**
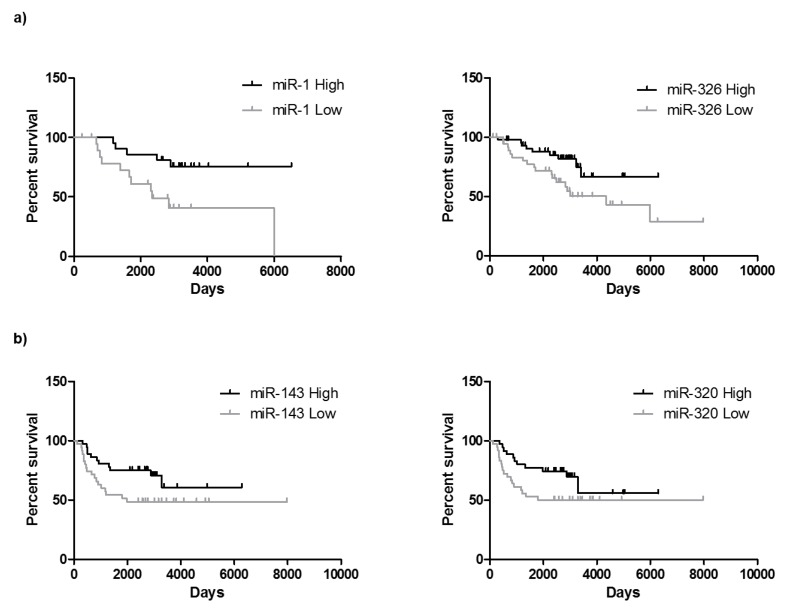
CRC progression indicated by miRNAs expression. Overall survival (**a**) and progression free survival (**b**) correlation studies have been performed by means of Kaplan Meier curves. Median value of each miRNA has been used to distribute high and low miRNA values. High expression of miR-1-3p and miR-326 are significantly associated with longer overall survival and higher expression of miR143-3p and miR-320a correlate with longer progression free survival.

As it can be observed in Figure 5a, the expression levels of 2 out of 14 miRNAs, i.e., miR-1 and miR-326 associate with patient’s overall survival. Indeed, higher levels of miR-1 and miR-326 were significantly associated with better overall survival. These results agree with the one described in Figure 4, since lower levels of miR-326 correlate with lymph node invasion and higher TNM. Moreover, the levels of miR-143-3p and miR-320a associate with progression free survival (Figure 5b). Indeed, higher levels of miR-320a were significantly associated with non-invasion of lymph nodes as shown in Figure 4.

### 2.7. Functional Significance of miRNAs

Since miRNA expression correlates with clinical features, we investigated the functional significance of the 13 validated miRNAs using bioinformatics approaches. For that purpose, we organized the miRNAs into 3 groups based on their relationship with age and Lynch syndrome, histopathological features and progression. We established a functional relation between miRNAs, based on shared targets, shown as a network: The purple colour in nodes indicates 3 or more miRNAs controlling the same target, dark purple indicates the maximum connection grade between miRNAs. Additionally, the shared targets were also grouped by most representative GO terms for function enrichment analysis. Age and Lynch syndrome, histopathological features and progression categories helped us to organize the data.

#### 2.7.1. Age and Lynch Syndrome

**Figure 6:**
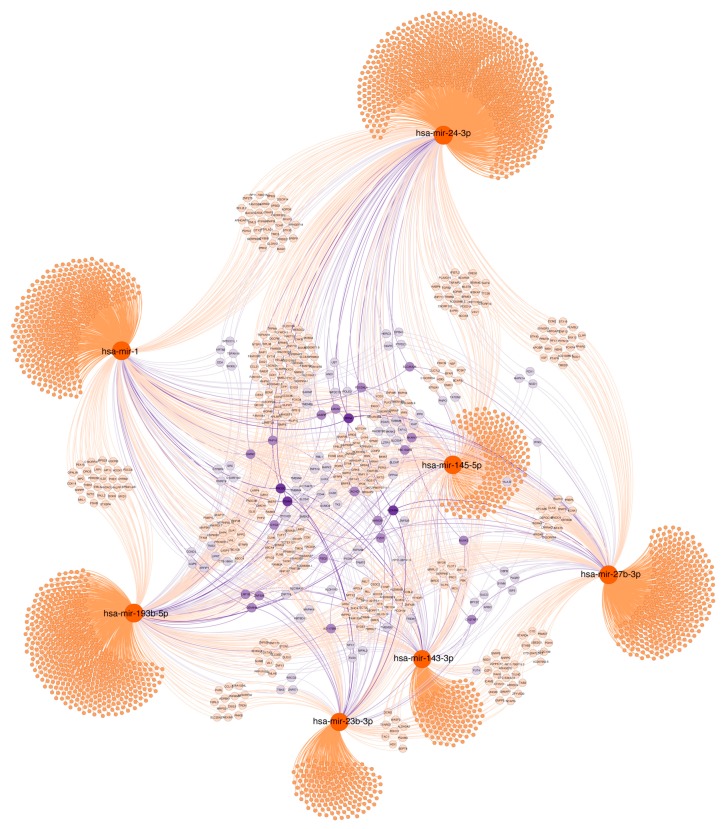
Target gene networks of validated miRNAs associated with age and Lynch syndrome. Selected miRNAs are highlighted in orange. Genes with at least two potential interactions were labelled. Genes with just one connection are unlabelled. Colour nodes of regulated genes are proportional to node degree (number of connections).

As shown in Figure 6, miR-1-3p, miR-23b-3p, miR-24-3p, miR-27b-3p, miR-143-3p, miR145-5p and miR193b-5p control the expression of several targets and appear to be related through genes including ALDH5A1, SLC25A25, FOXD4L4, or GABRP among others, which are relevant for metabolism, epigenetic regulation and migration. 

Moreover, function enrichment analysis (Supplementary Figure S1) shows that these miRNAs and their targets are involved in relevant biological processes including regulation of metabolic processes, cell proliferation and MAPK pathway-related functions, among others. Regarding cellular component, cell adhesion structures as well as Golgi function appears relevant. At the molecular functional level, kinase regulation and enzyme activity are enriched.

#### 2.7.2. Histopathological Features

**Figure 7:**
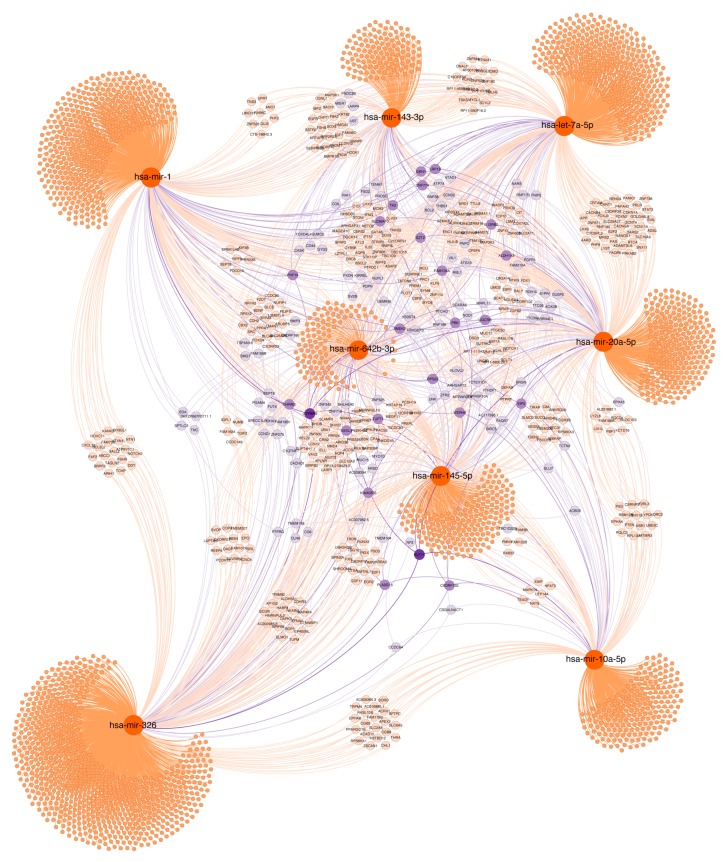
Target gene network of validated miRNAs associated with biopsy histopathological features. Selected miRNAs are highlighted in orange. Genes with at least two potential interactions were labelled. Genes with just one connection are unlabelled. Colour nodes of regulated genes are proportional to node degree (number of connections).

As shown in Figure 7, miR-1, miR10a-5p, miR-20a-5p, miR-145, miR-143, miR-326, miR-642b and Let7a-5p control the expression of several common targets including GTPBP4, KLF11, ALDH1A3, IL12RB2, BLC2, BMP3, CD44, ARGAP12, FGFR, MUC15, and SERPIN8, which are involved in relevant cell functions such as: angiogenesis, apoptosis, oxidative stress, cell trafficking and secretion or epithelial differentiation maintenance. These targets are linked to transcription factor and kinase activity, as molecular function analysis indicates. Adherent junctions, actin cytoskeleton and migration showed enrichment in the cellular components analysis. These functional analyses are presented in Supplementary Figure S2.

#### 2.7.3. Tumour Progression

**Figure 8:**
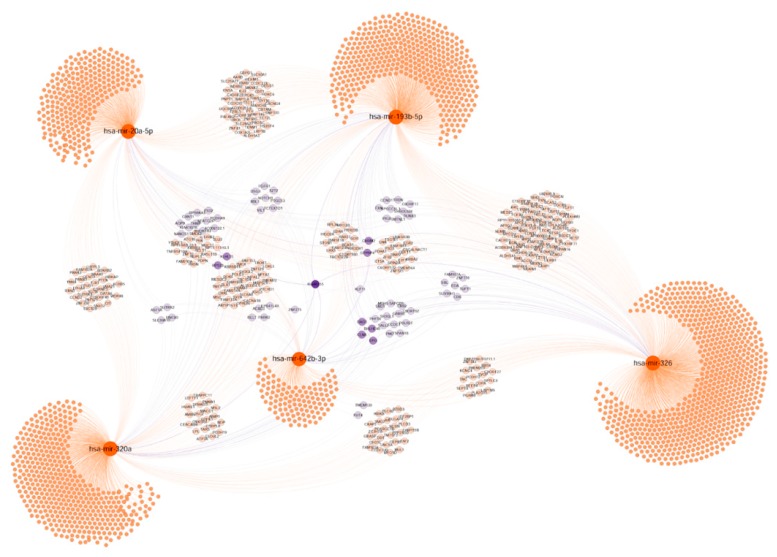
Target genes network of validated miRNAs associated with tumour invasion and progression. Selected miRNAs are highlighted. Genes with at least two potential interactions were labelled. Genes with just one connection are unlabelled. Colour nodes of regulated genes are proportional to node degree (number of connections).

For this group, including miR-20a-5p, miR-193b-5p, miR-320a, miR-326p and miR-342b-3p, predicted common target genes included: KIAA0355 (unknown function), CLN6, GTPBP4, EPO, KLF11, RBL1, ALDH5A1, CRB2, FGFR1, IGF1R, IL12Rb2, MAPK4K4, NOTCH1, and SERPIN8 (Figure 8). The bioinformatic analysis presented in Supplementary Figure S3 suggests that several of the predicted targets, including the above mentioned, are involved in cell functions related with tumour progression such as chemotaxis, focal adhesion, motility as well as apoptosis, proliferation and differentiation. Accordingly, the predicted targets are related with membrane biology and anchoring functions as the cellular component analysis indicated. Regarding molecular functions, receptor binding, protein ubiquitination and transcription factor binding were enriched. 

All these analyses strongly suggest that the validated miRNAs could function co-ordinately in CRC development and progression, regulating common target genes.

### 2.8. miRNAs Expression Could Regroup CRC Patients

**Figure 9:**
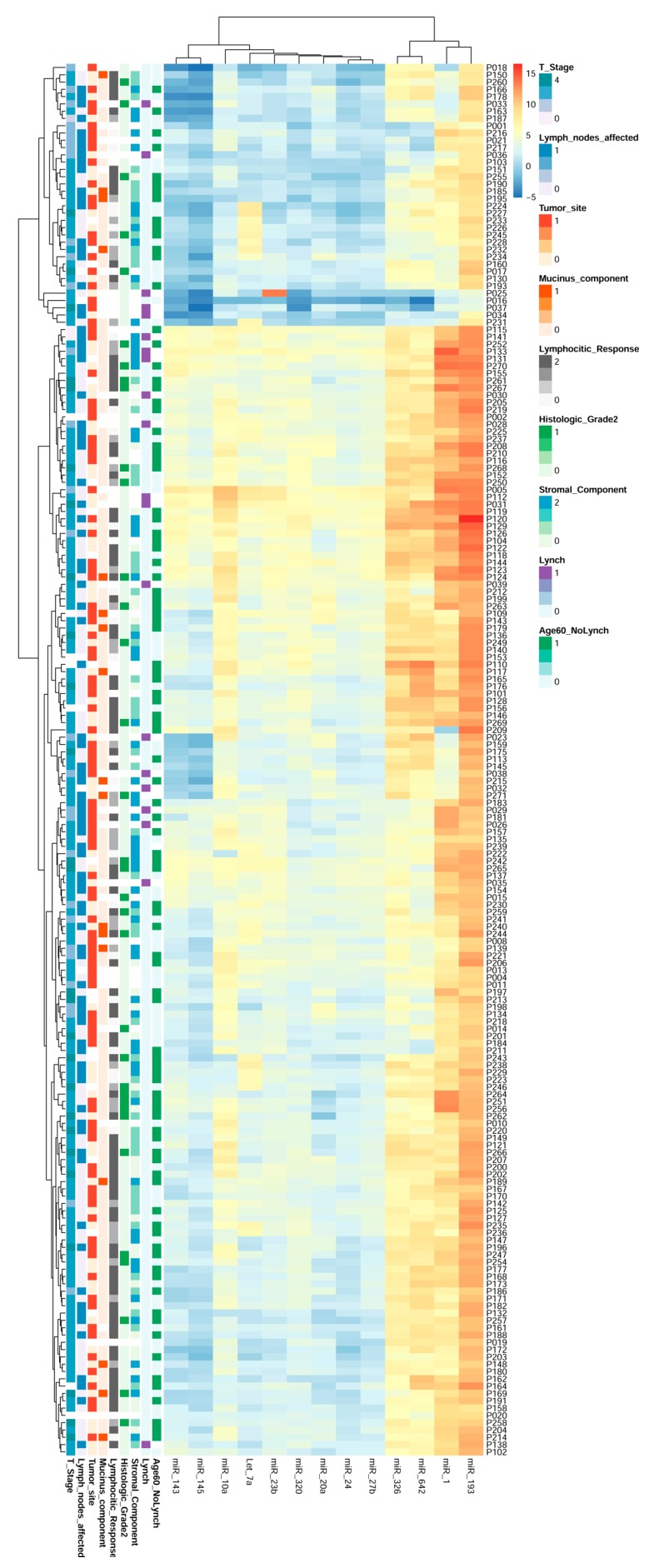
Heat map and hierarchical cluster of the validated miRNAs reclassifying CRC patients. The 13 validated miRNAs analysed by qRT-PCR in 192 CRC human biopsies allows for a new classification of the patients into 2 groups, which is independent of clinical features and based on miRNA pattern expression.

In order to further assess the utility of miRNAs present in CRC biopsies, additional bioinformatics analyses were performed. In particular we built a hierarchical cluster with the 13 miRNAs, which exhibited correlations with the studied clinical features. The results are shown in Figure 9.

As can be observed, the expression of the 13 validated miRNAs organized the patients into 2 groups: p18-p231 and p115-p102. These groups share miRNAs expression patterns independent of their clinical and histopathological characteristics, unveiling new molecular information that might be clinically useful and relevant.

On the other hand, the hierarchical clusters indicated that these 13 miRNAs also exhibited different grades of relation among them: miR-1, miR-93, miR642 and miR326 were more related, exhibiting a similar tendency in all patients but having lower relation to the rest of the miRNAs that clustered in the other group: miR-10a, miR-143, miR-145, Let-7a, miR- 23b, miR-320, miR-20a, miR-24 and miR-27b. But remarkably, the relationship exhibited between miRNAs strongly suggests that they regulate common pathways acting co-ordinately in CRC promotion and progression.

All these analyses indicate that the expression of miRNAs in biopsies clusters CRC patients in a manner independent of clinical data, regrouping the patients based on molecular markers, which may have potential clinical implications for patient management.

## 3. Discussion

CRC is a multifaceted disease due to the complex molecular mechanisms involved in its development and progression. Exhaustive investigations have been carried out in recent years to try to better characterize CRC tumours based on molecular profiles, with the final aim of offering more efficient management to the patients. Our work has identified, by hybridization arrays, and validated, by qRT-PCR, a combination of 13 miRNAs in 192 primary tumour biopsies with potential clinical relevance. Some of these miRNAs have not been previously related with CRC and they appear associated with relevant patient clinical characteristics including tumour progression, histopathological features such as differentiation grade, abundance of stroma, peritumoral inflammatory infiltrates, Lynch syndrome and age at diagnosis, and with overall survival as well as progression free survival. Moreover, by bioinformatic analysis, we predicted the functionality and the relevance of these miRNAs in the context of CRC, based on their predicted targets. Finally, we report that the expression of these miRNAs could contribute to a new classification of patient tumours with potential clinical usefulness.

Interestingly, our results demonstrate that only 2 miRNAs, miR-23b-3p y miR-24-3p were differentially expressed between Lynch syndrome and sporadic cases. Both miRNAs have not been previously related with this syndrome [19]. Moreover, hyper-methylation of miR132 has been associated to Lynch CRC [20]. miR-24 has been linked to bad CRC prognosis [21], and miR-24 regulates TRIM11, an E3 ubiquitin ligase with oncogenic properties promoting cell proliferation and inhibiting cell apoptosis [22]. Accordingly, our results indicate that mir24-3p is downregulated in Lynch tumours and may contribute to a bad CRC prognosis. In fact, TRIM (Tripartite Motif Family) members appear in our bioinformatic target prediction studies. miR-23b-3p has not been widely related with cancer, nor with colon cancer, although it has been predicted to be a regulator of MACC1 protein (metastasis-associated in colon cancer 1) [23]. Lynch syndrome is associated with satellite instability and DNA repair defects leading to tumour development.

Considering that Lynch syndrome is mainly diagnosed at a young age, we also studied whether any of our miRNAs could be associated with age. Our results demonstrate that miR-1-3p and miR193b-5p can discriminate between sporadic CRC diagnosed in patients less than 50 years of age and over 50 years old. However, this only explains a minority of young-onset CRC cases. Additionally, there is evidence strongly suggesting that young-onset CRC have a different molecular profile than late-onset CRC, although mechanisms underlying this difference are still unknown. Our results demonstrate, for the first time, that differences in miRNAs profiles are associated with diagnosis age in CRC. In fact miR-1-3p has been related with age in the smooth muscle of anal sphincter [24], age associated atrial fibrillation [25], and muscle integrity [26], but it has not been previously associated with CRC. MiR-1-3p belongs to the miR1/miR133 cluster and is considered to be a tumour suppressor, moreover it can also predict mortality in colon cancer in agreement with our results demonstrating a correlation of miR-1-3p with overall survival. It regulates prothymosin-α (PTMA) and purine nucleoside phosphorylase (PNP) in bladder cancer [27] and PIK3CA in NSCLC (Non-small cell lung carcinoma) [28]. miR-193b-5p has been related with cartilage aging [29]. In the context of CRC, mir193b-5p has been found to be deregulated compared to normal tissue [30]. It also promotes proliferation in SW620 cells, by affecting the expression of Smad3 and TGF-β [31]. Other miRNAs, including miR23b-3p, miR27b-3p, miR143-3p and miR145-5p, have been found to be associated to CRC diagnosis in patients over 60 years of age in our work. Our results shed light into the molecular mechanisms of young-onset CRC and might be useful for specific screening and management strategies in young CRC patients vs. older CRC patients.

Our results demonstrated that miR-1, miR10a-5p, miR-20a-5p, miR-145, miR-143, miR-326, miR-642b and Let7a-5p are correlated with relevant histopathological features including stroma abundance, tumour grade, peritumoral inflammatory infiltrates, mucin type and tumour location. It has been reported that the presence of peritumoral inflammatory infiltrates is negatively correlated with TNM leading to a better patient prognosis [32], although no molecular link has been unveiled. Our data also indicate that miR-642 is upregulated in tumours exhibiting large inflammatory infiltrates correlating with lower T stage or TNM classification, identifying for the first time this miRNA in CRC and linking both features relevant for patient prognosis. miR642b has been related to proliferation in the context of prostate cancer [33], bladder cancer [34] and pancreatic neuroendocrine tumours [35]. Additionally, it has been recently found in liver metastasis of CRC primary tumours [36]. It might contribute to the process of metastatic cell adaption to the liver microenvironment, indicating that primary and metastatic tumours also share miRNAs. In fact, miR-1 is considered to be a tumour suppressor by restraining Epithelial Mesenchymal Transition (EMT) in several cancers, including CRC [37]. Moreover, miR-20a-5p, miR-193-3p, miR-320a, miR-326, miR-642b-3p have also been associated to stage (low stage I–II and high stage III–IV) and invasiveness (T2–T4) in primary CRC biopsies [38]. Accordingly, we demonstrated that miR-326 correlated with longer overall survival in our patient cohort. miR-20a-5p is also linked to CRC and it has been reported as an independent progression marker in CRC, since it correlates with lymph node invasion in primary tumours [39] and in faeces after tumour resection [40]. miR-10a-5p has been recently associated to suppression of CRC metastasis by modulating EMT and cell death [41]. miR-326a is also considered to be a tumour suppressor and it has been associated with better CRC prognosis, corresponding with our results. This miRNA inhibits tumour proliferation and metastasis [42]. Moreover, our results demonstrate that miR-326a is associated to tumour location and it is downregulated in right-side tumours. Since miR-326a is considered a tumour suppressor, this downregulation could contribute to a worse prognosis of right-side tumours. It might cooperate with miR-146a and miR-147b, which are also downregulated in right-side tumours, as previously reported [43]. Our results highlight the potential use of miRNA analysis in primary tumours as markers of critical cell processes such as EMT and invasion, and therefore as markers of CRC progression. In fact, 3 out of the 5 miRNAs identified in this work were associated with tumour progression: miR-20a-5p, miR-193b-5p, miR-320a, miR-326p and miR-642b-3p, were previously linked to invasion and metastasis. miR-193-5p controls CRC cell proliferation and invasion, by regulating the TGF-β pathway [31] and Stathmin 1 [44]. miR-320 has been included in the CRC liver metastasis miRNA signature [45] regulating neuropilin 1 (NRP-1) among other pro-invasion genes [46], and Rac 1 [47]. miR-642b-3p is the best predictor of regional lymph node metastasis in endoscopically resected T1-stage, regulating critical genes such as E2F1, RAP2B, and AKT1 [48].

Previous studies have reported a consensual classification of CRC patients into 4 different subtypes based on cellular and molecular characteristics of the primary tumours [6]. In this classification, based on analysis of 255 biopsies, 110 miRNAs were differentially expressed between the subtypes. The CMS2 subtype, including its chromosome instability, among other features, includes upregulation of the mir-17–92 cluster, regulating CMYC. Our results indicate that Lynch syndrome involves the dysregulation of miR-23b-3p and miR-24-3p, linking two novel miRNAs with MMR defects. In CMS3, an epithelial subtype exhibiting RAS mutation, hsa-mir-143 and miRNAs belonging to the let-7 family are involved. CMS4 is enriched in downregulated miRNAs including hsa-mir148a, the miR-192 and miR-200 families. The miR-200 family regulates EMT pathways by targeting ZEB1 and/or ZEB2 [49], whereas hsa-mir-148a is predicted to regulate MMP13 and TGFB2, which are important for the extracellular matrix remodelling and the TGF-β pathway. Taken together, the downregulation of miRNAs associated with suppression of EMT-, MR- and TGF-β-associated signatures could explain why CMS4 is the most aggressive subtype and exhibits the poorest prognosis.

A precise classification of CRC patients based on biological and molecular characteristics of tumours is essential for an efficient and personalized CRC management, particularly treatment election. Our results unveil combinations of miRNAs in primary CR tumours associated with age, histopathological features of the biopsy, tumour progression and other clinical patient data, allowing new stratification of patients that might be useful for CRC treatment election. The miRNAs validated here, among others, could also be considered as novel biomarkers of CRC subpopulations and possibly accurate biomarkers for CRC treatment response in clinical practice as has been recently suggested [50,51,52]. Related to this, biological significance of the validated miRNAs has been also addressed using bioinformatics and based on miRNAs target predictions. GO terms enrichment confirmed that most of these miRNAs interact modulating CRC development and progression. However, functional assays should be performed in order to demonstrate this critical point, considered as a limitation of the present study.

A group of selective and accurate molecular CRC biomarkers, including our miRNAs, easily detected by qRT-PCR, would constitute robust tools for affordable personalized medicine implementation.

## 4. Material and Methods

### 4.1. Colorectal Cancer Biopsies Collection: Ethics Statement

A total of 200 CRC tissue samples were collected from patients who underwent CRC surgery at the Ramon y Cajal University Hospital, Elche University Hospital and Virgen de la Victoria University Hospital. Biopsies were evaluated by a pathologist and staged according to the guidelines of the Union for International Cancer Control tumour-node-metastasis TNM staging system (AJCC/UICC TNM, 7th edition). The ethics committee of the Ramón y Cajal University Hospital approved this study. The internal code of the ethics committee of the Ramón y Cajal University Hospital approval for our study is 011/11 and the permission date was 29 March 2011. Tissue was obtained from paraffin-embedded biopsies via manually punches from tumour regions selected by the pathologist.

### 4.2. microRNA Array Profiling

Hybridization Array screening of 1436 miRNAs was performed at Exiqon Services (Vedbaek, Denmark). The samples used for profiling assay are indicated in Table 1. Quality of total RNA was verified by an Agilent 2100 Bioanalyzer. We used 750 ng of total RNA from each sample and the reference was labelled with Hy3™ and Hy5™ fluorescent tags, respectively, using the miRCURY LNA™ microRNA Hi-Power Labeling Kit. The Hy3™-labelled samples and a Hy5™-labelled reference RNA sample were mixed pair-wise and hybridized to the mercury LNA™ microRNA Array 6th Gen instructions, using a Tecan HS4800™ hybridization station (Tecan, Grödig, Austria). After hybridization, the microarray slides were scanned. The miRCURY LNA™ microRNA Array slides were scanned using the Agilent G2565BA Microarray Scanner System (Agilent Technologies, Inc., Santa Clara, CA, USA). The quantified signals were background corrected (Normexp with offset value 10 [53]) and normalized using a quantile normalization method.

52 different Spike-in controls were added in various concentrations in both the Hy3™ and the Hy5™ labelling reactions for evaluating the labelling reaction, hybridization, and the performance of the array experiment. Each spike-in control had 4 replicates of capture probes on the array.

The background threshold was calculated for each individual microarray slide. miRNAs exhibiting signal intensity above this threshold in less than 20% (or 2) of the samples were removed.

The miRNA profiling identified a subset of 200 top miRNAs (miRNAs with absolute value of the log fold change larger than 1) out of the total number analysed (492) that are differentially expressed in the different samples. 

For final selection, miRNAs showing most prominent and significant changes were also functionally analysed, based on their predicted target genes. For each miRNA, potential targets were downloaded from the Targetscan Human 5.1 database (http://www.targetscan.org/vert_50/). Target gene list were then evaluated using the online Bioinformatic Database for Annotation, Visualization and Integrated Discovery (DAVID) (http://david.abcc.ncifcrf.gov/tools.jsp). Only miRNAs having target genes enriched in functional categories relevant for CRC were included in the panel for further validation (Table 1).

### 4.3. Validation of Selected miRNAs: Total RNA Extraction and RT-qPCR

Punches obtained from paraffin-embedded tumour biopsies were crushed to favour tissue digestion. Total RNA enriched in miRNAs was extracted with miRNeasy FFPE kit (Qiagen, 217504; Hilden, Germany), following the manufacturer’s indications.

miRNAs were determined and quantified by RT-qPCR using the Universal RT miRNA PCR System (Exiqon, Vedbaek, Denmark). Briefly, 200ng of RNA was used as template for RT in a final volume of 20 µL. cDNA was diluted 1/11 with nuclease-free sterile water and 4 µL were used as template for PCR.

PCR detection was performed using SYBR Green and specific LNA probes for each selected miRNA (Exiqon). All reactions were carried out in triplicate using a Light Cycler 480 instrument (Roche, Basel, Switzerland) and Cq values were calculated using a 2nd derivative method (Light Cycler 480 Software 1.5, Roche). miRNA expression values are presented as ΔCq, obtained as follows: ΔCq = miRNA Cq − housekeeping Cq. We use the mean of 5S Cq and RNU6B Cq as housekeeping Cq.

### 4.4. Bioinformatics Studies

Predicted target genes of selected miRNAs were recovered from miRGate database. Targets genes were selected using as criteria that at least had two positive computational predictions with different algorithms or reported biological evidence. Functional enrichment analysis was performed using GOstats (Gene Ontology Statistics). A threshold of *p*-value < 0.001 was selected to filter significant GO terms [54]. Bipartite networks were built using the selected miRNAs as source and putative regulated genes as targets. Networks were represented using the ForceAtlas2 layout algorithm implemented in Gephi [55].

### 4.5. Statistical Analysis

The normal distribution of variables were assessed with the Shapiro-Wilk test. For normal distributed data, *t*-test and ANOVA with post hoc Bonferroni correction for multiple comparisons were used after assessing homogeneity of variances with the Levene test. For group comparison of non-normal distributed data, the Kruskal-Wallis test was performed. Intergroup differences of non-normal data were assessed with post hoc Mann-Whitney U-tests. Overall survival (OS) curves were calculated by the Kaplan-Meier method and the log-rank test was used to determine the difference in OS rates between two groups. The median value of miRNA expression in all samples was chosen as the cut-off point for separating low- and high-level expression of miRNAs. Statistical Package for the Social Sciences (SPSS) software version 19.0. was used for the analysis.

## 5. Conclusions

In this work, we demonstrate that miRNAs associated to CR primary tumour histopathological features could be helpful for a better tumour characterization and more accurate patient prognosis in CRC. Validated miRNAs may re-classify CRC patients more accurately, based on their transcriptomic characteristics. Moreover, these miRNAs would act co-ordinately through common gene targets involved in CRC development and progression pathways, shedding light on molecular mechanisms underlying tumour development. In particular, miRNAs associated to tumour stage and regional lymph node infiltration could be considered as accurate biomarkers of CRC progression. Finally, different miRNA profiles could also be found associated with patient age at the time of diagnosis and with Lynch syndrome.

In summary, all these results demonstrate that miRNA detection in primary CR tumours would allow for personalized patient management.

## Figures and Tables

**Table 1:** Clinical features of patients using in screening analyses. Age, staging, tumour side and histological grade are shown. (NA) not applicable; (-) not available data.

Patient	Group	Age	Staging	Tumour Site	Histologic Grade
1	Control	51	NA	NA	NA
2	<45 Non Lynch	27	IIA	Left	-
3	<45 Non Lynch	35	IIA	Right	Well differentiated
4	Lynch	46	-	-	Moderately differentiated
5	Lynch	47	IIIB	Transverse	Moderately differentiated
6	Lynch	41	IIA	Left	Well differentiated
7	>45 Non Lynch	70	IVA	Left	Well differentiated
8	>45 Non Lynch	64	IIIB	Left	Well differentiated

**Table 2:** Selected miRNAs for further validation in 192 paraffin-embedded tumour biopsies. miRNA accession numbers and absolute values of the log of fold change in comparison to the control biopsy are presented.

miRNA ID	Accession Number	<45 Non Lynch	Lynch	>45 Non Lynch
		logFC	logFC	logFC
**hsa-miR-1-3p**	MIMAT0000416	0.02	−0.22	3.13
hsa-miR-10a-5p	MIMAT0000253	−0.43	−0.50	2.55
hsa-miR-16-5p	MIMAT0000069	1.97	2.99	4.47
hsa-miR-20a-5p	MIMAT0000075	0.71	1.83	3.66
hsa-miR-23b-3p	MIMAT0000418	2.83	2.96	6.05
hsa-miR-24-3p	MIMAT0000080	1.82	2.31	5.15
hsa-miR-27b-3p	MIMAT0000419	0.68	1.07	3.93
hsa-miR-29c-3p	MIMAT0000681	1.29	1.92	4.16
hsa-miR-99a-5p	MIMAT0000097	−0.54	−1.46	0.38
hsa-miR-143-3p	MIMAT0000435	0.17	−0.35	4.59
hsa-miR-145-5p	MIMAT0000437	−0.03	−0.02	4.45
hsa-miR-193b-5p	MIMAT0004767	−0.23	−0.59	0.36
hsa-miR-320a	MIMAT0000510	−1.68	−1.60	0.05
hsa-miR-326	MIMAT0000756	−1.38	−1.89	−2.89
hsa-miR-642b-3p	MIMAT0018444	−1.46	−1.57	−3.05
hsa-Let7a-5p	MIMAT0000062	1.14	1.58	3.68
SNORD4A	NR_000010.1	−0.42	1.00	2.10

**Table 3:** Clinical data for the patients and biopsies used in the miRNA validations. Data regarding age, gender, tumour site, T-stage, N-stage, M-stage and staging according to AJCC 7th edition are shown.

Characteristics	Patients
Gender (m:f) absolute numbers	115:77
Lynch syndrome	
Lynch	20
Sporadic	170
Age, years at diagnosis	
Under 50	59
Over 50	132
Under 60	87
Over 60	103
Tumour site	
Right	71
Left	101
Unknown	20
T stage	
Tis	2
T1	1
T2	29
T3	126
T4	27
Tx	7
N stage	
N0	80
N1	44
N2	58
Nx	10
M stage	
M0	166
M1	16
NS/NC	10
AJCC staging 7th ed	
Stage 0	2
Stage I	25
Stage IIA	50
Stage IIB	4
Stage IIC	2
Stage IIIA	3
Stage IIIB	53
Stage IIIC	29
Stage IVA	13
Stage IVB	3

**Table 4:** Histopathological data for the biopsies used in the miRNA validations. Data regarding histological grade, stroma abundance, mucinous component, peritumoral inflammatory infiltrate are presented.

Characteristics	Patients
Histologic grade	
Well differentiated	92
Moderately differentiated	54
Poorly differentiated	35
Undifferentiated	0
Unknown	11
Stroma	
Abundant	33
Moderate	57
Poor	59
Unknown	43
Mucinus component	
Mucinus	17
No mucinus	142
Unknown	33
Peritumoral inflammatory infiltrate	
Marked	24
Moderate	40
Poor	86
Unknown	42

## Supplementary Materials

The following are available online at https://www.mdpi.com/2072-6694/11/3/346/s1, Figure S1: Functional enrichment analysis of miRNAs associated with age and Lynch syndrome, Figure S2: Functional enrichment analysis of miRNAs associated with biopsy histopathological features, Figure S3: Functional enrichment analysis of miRNAs associated with tumour invasion and progression.

## Transcript Profiling

Accession number of GEO repository for expression microarray data: GSE123040.

## Abbreviations

CRC	Colorectal cancer
MiRNAs	microRNAs
CIN	Chromosomal instability
MSI	Microsatellite instability
CIMP	CpG island methylator phenotype
MMR	DNA mismatch repair
CMS	Consensus molecular subtype
TNM	Tumour-node-metastasis
FFPE	Formalin-Fixed Paraffin-Embedded
LNA	Locked Nucleic Acid
Cq	quantitation cycle
GO	Gene ontology
NSCLC	Non-small-cell lung carcinoma
EMT	Epithelial to mesenchymal transition
BP	Biological process
CC	Cellular component
MF	Molecular function
