# Supplementary material for: Novel Molecular Characterization of Colorectal Primary Tumors Based on miRNAs
Source: Cancers (Basel). 2019 Mar 11;11(3):346. doi: 10.3390/cancers11030346 (PMC6468580; doi:10.3390/cancers11030346)
Supplement: Supplementary file 1 [file cancers-11-00346-s001.pdf]

Supplementary Materials: Novel Molecular Characterization of Colorectal Primary Tumors Based on miRNAs

Elisa Conde Moreno, Alejandro Pascual, Daniel Prieto-Cuadra, Val F. Lanza, Javier Molina-Cerrillo, Miren Edurne Ramos-Muñoz, Esperanza Macarena Rodríguez-Serrano, José Luis Soto, Alfredo Carrato, María Laura García-Bermejo and Carmen Guillen-Ponce

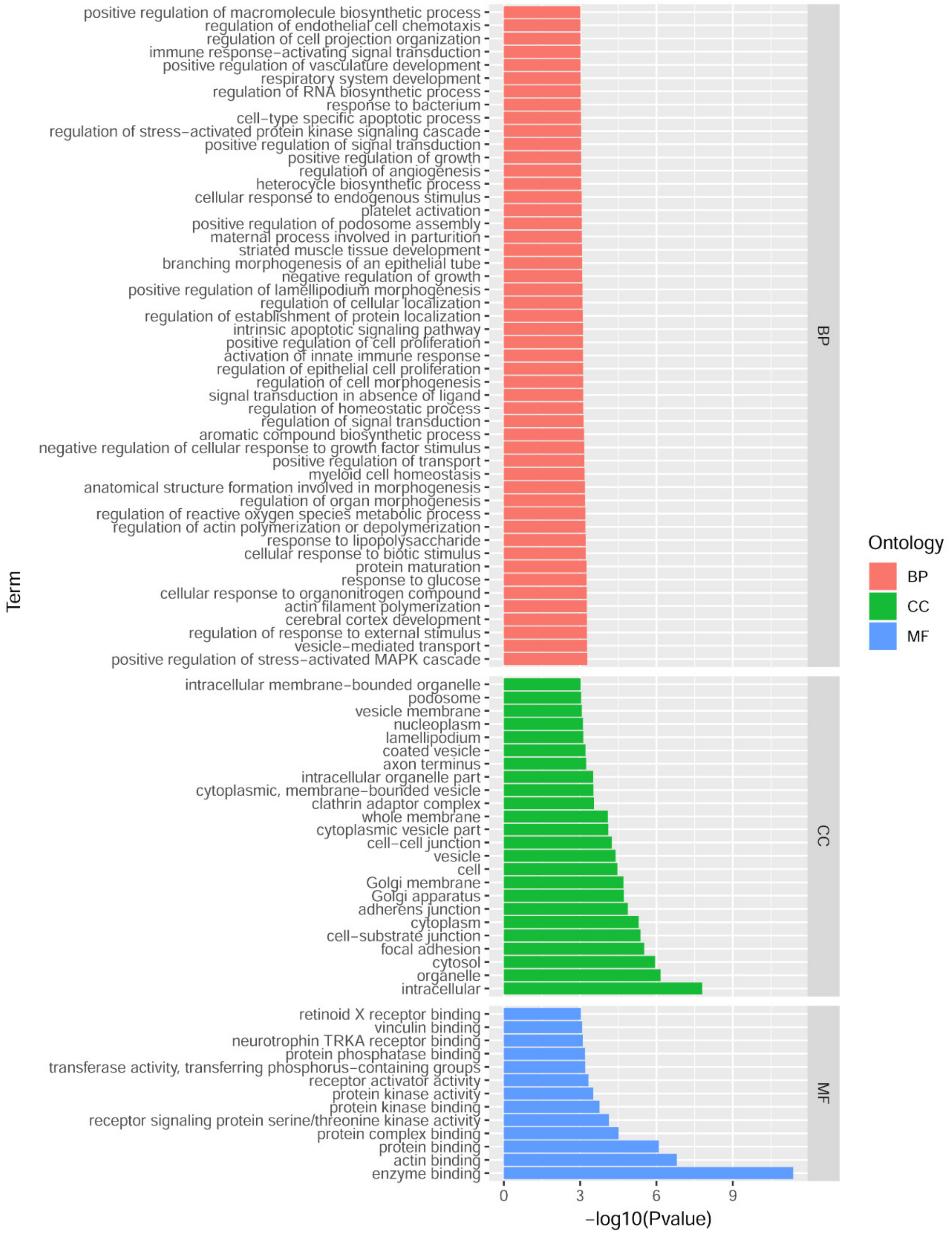

Figure S1. Functional enrichment analysis of miRNAs associated with age and Lynch syndrome. Bar plot of statistically significant GO terms. The three GO categories are divided: BP (Biological process) in red, CC (Cellular component) in green, MF (Molecular function) in blue.

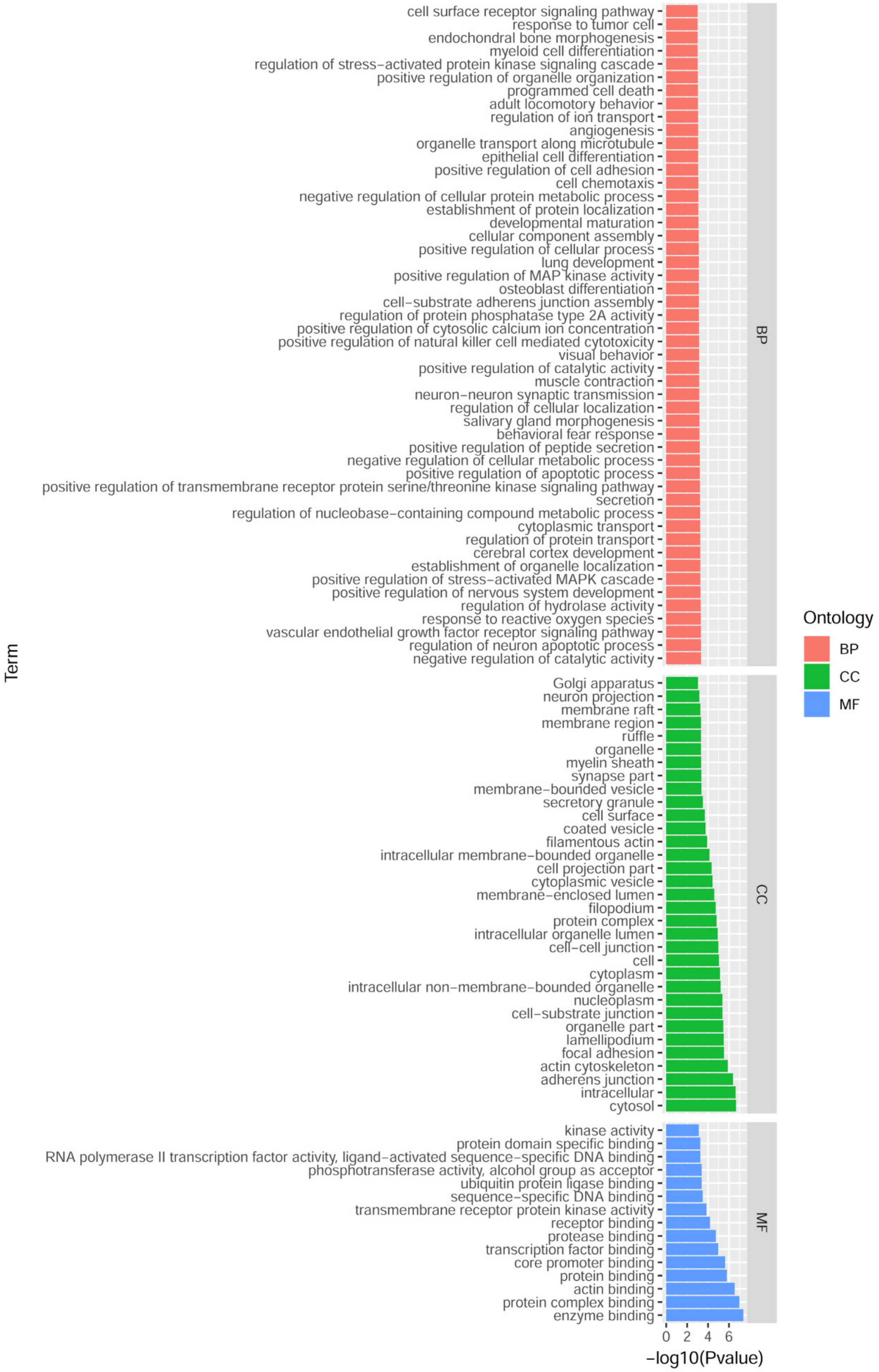

Figure S2. Functional enrichment analysis of miRNAs associated with biopsy histopathological features. Bar plot of statistically significant GO terms. The three GO categories are divided: BP (Biological process) in red, CC (Cellular component) in green, MF (Molecular function) in blue.

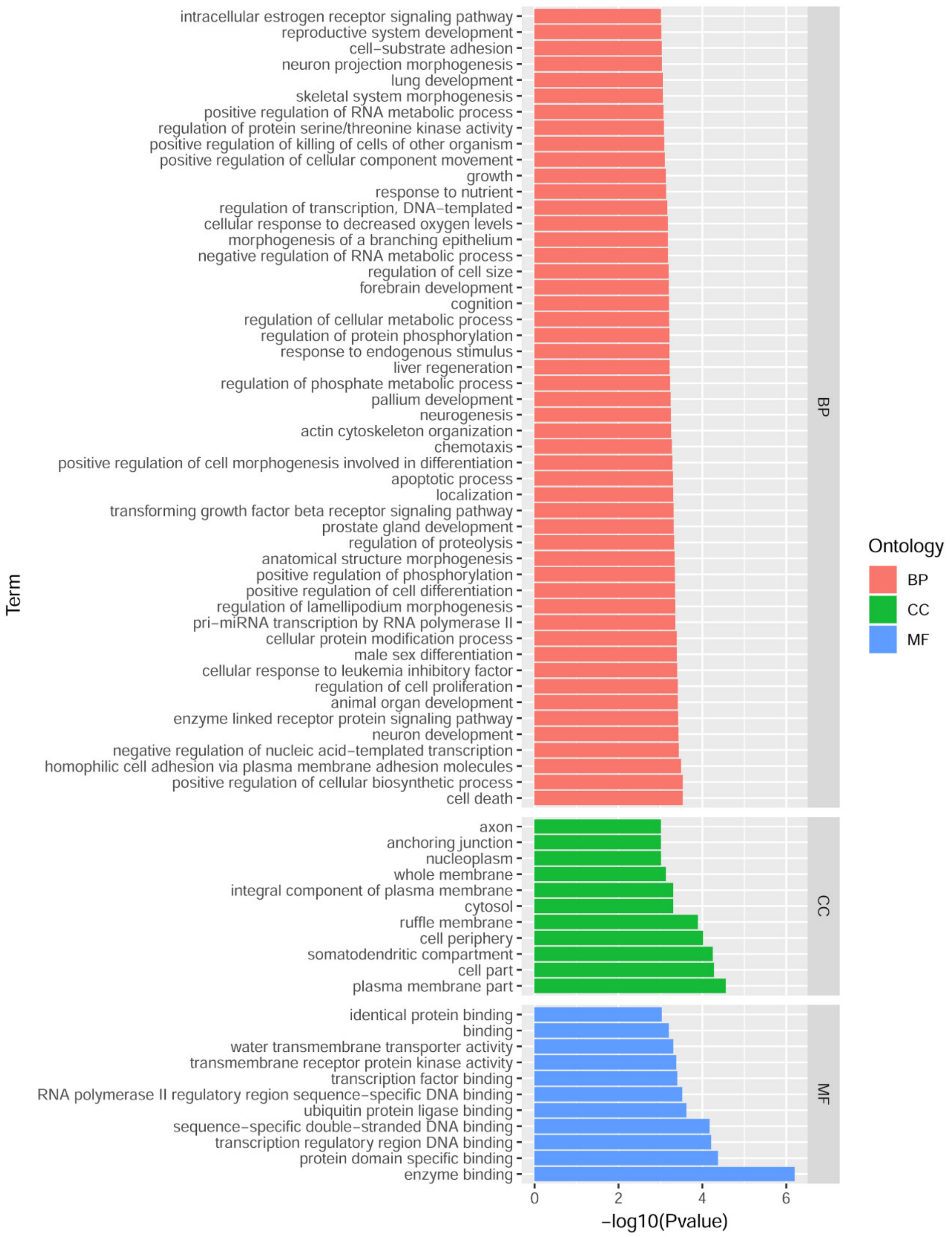

Figure S3. Functional enrichment analysis of miRNAs associated with tumor invasion and progression. Bar plot of statistically significant GO terms. The three GO categories are divided: BP (Biological process) in red, CC (Cellular component) in green, MF (Molecular function) in blue.

© 2019 by the authors. Licensee MDPI, Basel, Switzerland. This article is an open access article distributed under the terms and conditions of the Creative Commons Attribution (CC BY) license (http://creativecommons.org/licenses/by/4.0/).
